# Supplementary material for: The ribosome-associated complex RAC serves in a relay that directs nascent chains to Ssb
Source: Nat Commun. 2020 Mar 20;11:1504. doi: 10.1038/s41467-020-15313-w (PMC7083937; doi:10.1038/s41467-020-15313-w)
Supplement: Supplementary file 1 — Supplementary Information [file 41467_2020_15313_MOESM1_ESM.pdf]

Supplementary Information

The ribosome-associated complex RAC serves in a relay that directs nascent chains to Ssb

Ying Zhang *et al.*

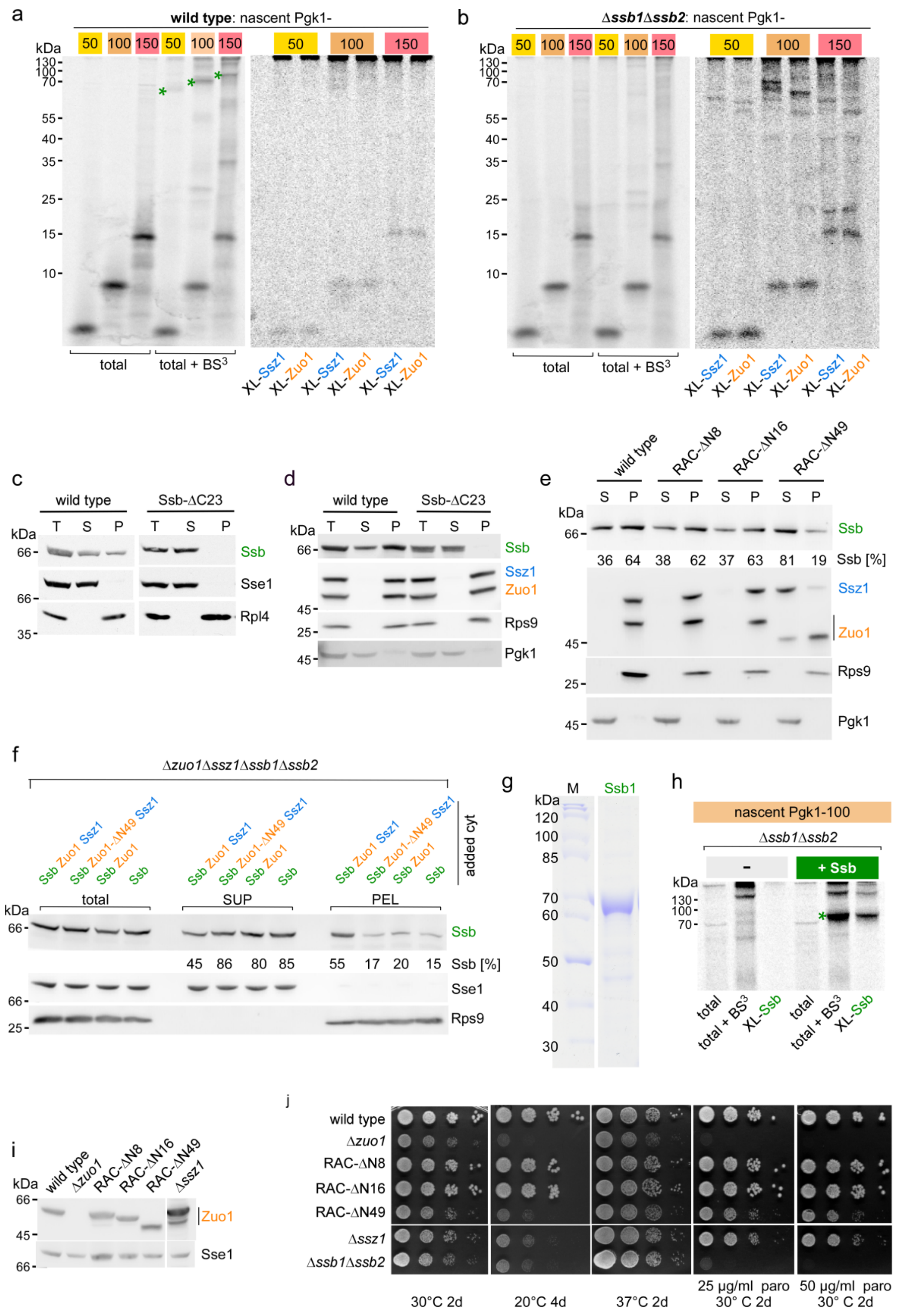

Supplementary Figure 1.

Properties of RAC variants containing N-terminal deletions within Zuo1.

(**a-b**) Contacts of Zuo1 and Ssz1 with 50-, 100, or 150-residue nascent Pgk1. RNCs were generated in a wild type (**a**) or Δ*ssb1*Δ*ssb2* translation extract (**b**) in the presence of [^{35}S]-methionine. Nascent chain crosslink products were isolated via immunoprecipitation with antibodies directed against Ssz1 (XL-Ssz1) or Zuo1 (XL-Zuo1). Crosslink products to Ssb, which can be detected in total extract, are labelled by green asterisks. Totals before and after (total +BS^3) crosslinking represent 5% of the immunoprecipitation input. Loading was normalized to the amount of nascent chains in the total prior to crosslinking.

(**c**) Ribosome binding of wild type Ssb and Ssb-ΔC23 in total cell extract. Total cell extract of wild type (mycSsb1) and Ssb-ΔC23 was separated into a cytosolic supernatant (S) and a ribosomal pellet (P) by ultracentrifugation. Aliquots of supernatant and pellet were analysed via immunoblotting with the antibodies indicated. Pgk1 served as a cytosolic marker and Rpl4 as a ribosomal marker. Ssb-ΔC23 was fully recovered in the cytosol. This observation is consistent with previous findings [1].

(**d**) Binding of Ssb to ribosomes/RNCs in a wild type or Ssb-ΔC23 translation extract. The experimental set-up was as described in **Fig. 1d**, however, instead of performing BS^3-crosslinking the total translation reaction (T) was separated into a cytosolic supernatant (S) and a ribosomal pellet (P) via ultracentrifugation. Aliquots were analysed on a Tris-Tricine gel followed by immunoblotting with the indicated antibodies. Pgk1 served as a cytosolic marker, Rps9 as a ribosomal marker. Ssb-ΔC23 was not ribosome-associated but fully recovered in the supernatant fraction.

(**e**) Ribosome binding of Ssb and mutant RAC versions. Total cell extract of wild type, RAC-ΔN8, RAC-ΔN16, or RAC-ΔN49 was separated into a cytosolic supernatant (S) and a ribosomal pellet (P) by ultracentrifugation. Aliquots of supernatant and pellet were analysed on a Tris-Tricine gel followed by immunoblotting with the indicated antibodies. Pgk1 served as a cytosolic marker and Rps9 as a ribosomal marker. Ssz1 was ribosome-bound in total extract of wild type, RAC-ΔN8, or RAC-ΔN16, however, was localized to the cytosol in RAC-ΔN49. About 2/3 of Ssb was ribosome-bound in wild type, RAC-ΔN8, or RAC-ΔN16. However, only about 1/3 of Ssb was ribosome-bound in cells expressing RAC-ΔN49. This observation is consistent with previous findings [1]. The distribution of Ssb between S and P is indicated below the immunoblot decorated with α-Ssb (Ssb [%]).

(**f**) Rebinding of Ssb to ribosomes/RNCs derived from a Δ*ssb1*Δ*ssb2*Δ*zuo1*Δ*ssz1* translation extract. The experimental set-up was as described in **Fig. 1e**. RNCs were generated in a Δ*zuo1*Δ*ssz1*Δ*ssb1*Δ*ssb2* translation extract. Ribosome-free extracts obtained from wild type (Ssb, Zuo1, Ssz1), Δ*zuo1* Zuo1-ΔN49 (Ssb, Zuo1-ΔN49, Ssz1), Δ*ssz1* (Ssb, Zuo1), Δ*zuo1*Δ*ssz1* (Ssb) were added after completion of translation reactions (total). Instead of performing BS^3-crosslinking as in **Fig. 1e**, the total was separated into a cytosolic supernatant (SUP) and a ribosomal pellet (PEL) via ultracentrifugation. Aliquots were analysed on a Tris-Tricine gel followed by immunoblotting with the indicated antibodies. Sse1 served as a cytosolic marker, Rps9 as a ribosomal marker. The distribution of Ssb between SUP and PEL is given as a percentage (Ssb [%]).

(**g**) Purified His_6-*Sc*Ssb1. Coomassie-stained gel showing 5 µg of purified Ssb, which was used for the experiments shown in **Fig. 1f** and **Supplementary Fig. 1h**.

(**h**) Purified Ssb added to a Δ*ssb1*Δ*ssb2* translation extract contacts ribosome-bound nascent chains. RNCs were generated in a Δ*ssb1*Δ*ssb2* translation extract (-). Purified Ssb1 was added as indicated (+ Ssb). Totals before and after crosslinking (total $+BS^3$) represent 5% of the material employed for immunoprecipitation reactions. Loading was normalized according to the signal of the [^{35}S]-labelled nascent chain in the total prior to crosslinking. The crosslink between nascent Pgk1-100 and Ssb in the total after crosslinking is indicated by a green asterisk. Immunoprecipitated crosslink product between Pgk1-100 and Ssb (XL-Ssb).

(**i**) Expression level of Zuo1 and various Zuo1 mutants in a Δ*zuo1* background. Total protein extracts of the indicated strains were analysed via immunoblotting with antibodies directed against Zuo1. The cytosolic protein Sse1 served as a loading control. All samples were run on a single Tris-Tricine gel. For detailed information about the strains see **Supplementary Table 1.**

(**j**) Phenotypic analysis of Zuo1 mutant strains. Log phase cultures of Δ*ssb1*Δ*ssb2* and strains as shown in **g** were spotted onto YPD plates, which were incubated at the indicated temperatures and times (see Supplementary Methods). Paromomycin (paro) was added at the concentration indicated. For each growth condition strains were analysed on a single plate. The growth analysis revealed that inefficient transfer from RAC to Ssb is not necessarily connected to the severe growth defects observed in the absence of Ssb, Zuo1, or Ssz1 [2,3]. This resembles previous observations. For example, strains expressing an Ssz1 mutant lacking the complete SBDβ suffer from only mild growth defects [4,5]. Likewise, Ssb mutants unable to bind to the ribosome as for example Ssb-ΔC23, do not cause significant growth defects *in vivo* [1,6]. Source data for **a-j** are provided as a Source Data file.

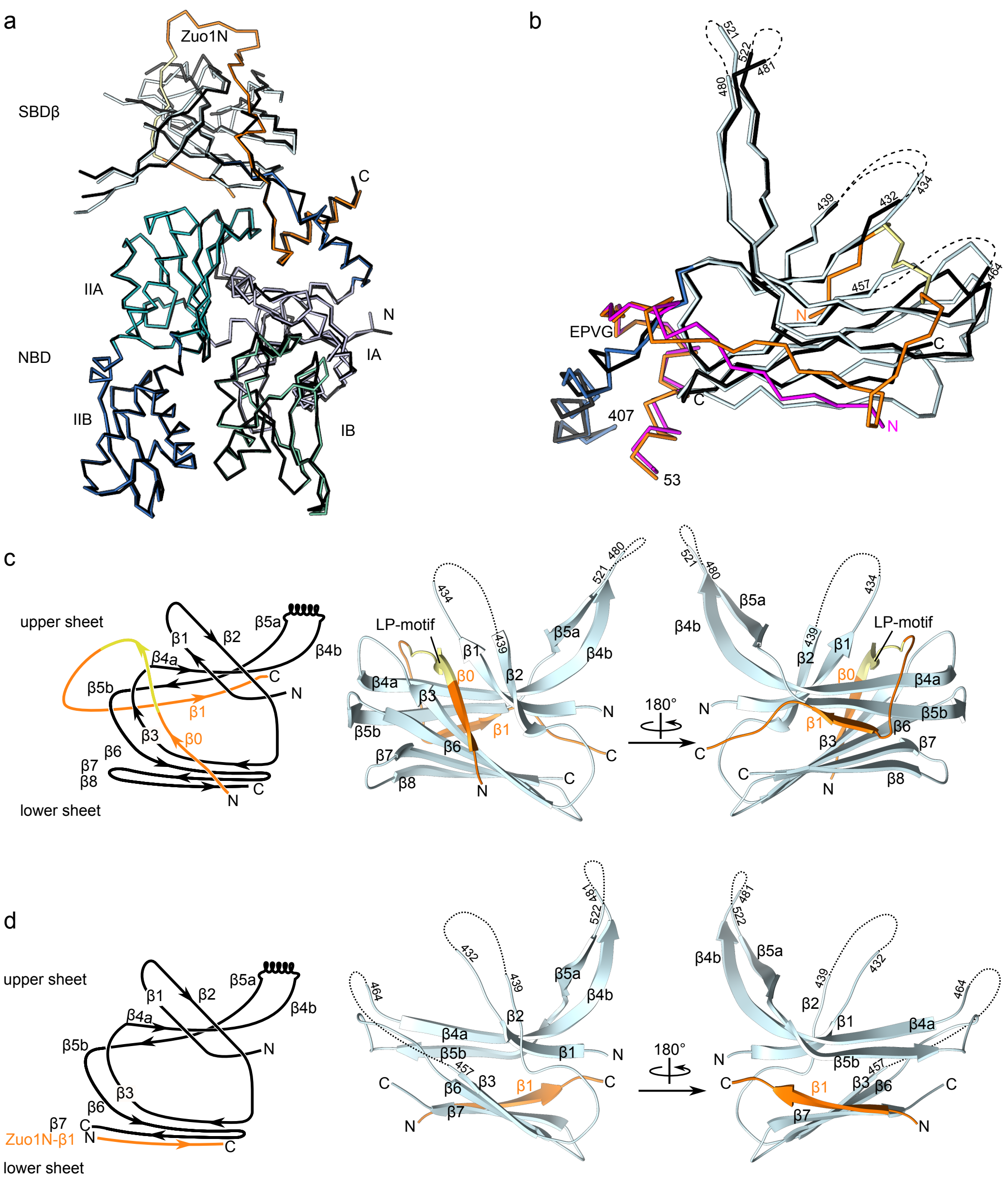

Supplementary Figure 2.

Comparison of the structures of Ssz1-Zuo1N and Ssz1-Zuo1N$^{19-60}$.

(**a**) Superposition of the Ssz1-Zuo1N structure (this study; colored as in **Fig. 2b**) with the previous Ssz1-Zuo1N$^{19-60}$ structure, which lacks the β0 and the LP-motif (Ssz1 and Zuo1N in black; PDB 5MB9, [7]). Only Cα atoms are shown. Both structures are highly similar indicated by a low r.m.s. deviation of 0.486 Å for 391 atom pairs in the Ssz1-NBD, 0.603 Å for 23 atom pairs in the Ssz1 inter-domain linker (αL, βL) - Zuo1N (β2 to α1) region, and 0.824 Å for 83 atom pairs in the Ssz1-SBDβ - Zuo1N (β0 to β1) region. The new Ssz1-Zuo1N structure contains two heterodimers in the asymmetric unit. The two heterodimers have an r.m.s.d. of 0.5 and 0.4 Å for 513 and 51 equivalent Cα atom positions for Ssz1 and Zuo1N, respectively.

(b) Close-up of the Ssz1-linker region, SBDβ and Zuo1N shown in **a.** Coloring is as in **a** for the new structure, and Ssz1 in black and Zuo1N in magenta for PDB 5MB9, [7].

(**c** and **d**) Details of the Ssz1-SBDβ structures from (**c**) Ssz1-Zuo1N (this study) and (**d**) Ssz1-Zuo1N$^{19-60}$ (PDB 5MB9, [7]). The left panels show a schematic representation of the topology of the β-sandwich domain. The middle and right panels show the structures in ribbon representation in two different orientations. Ssz1-SBDβ in cyan, Zuo1N in orange and the LP-motif in yellow. The left and middle panels are oriented the same way, while the right panels are rotated by 180°.

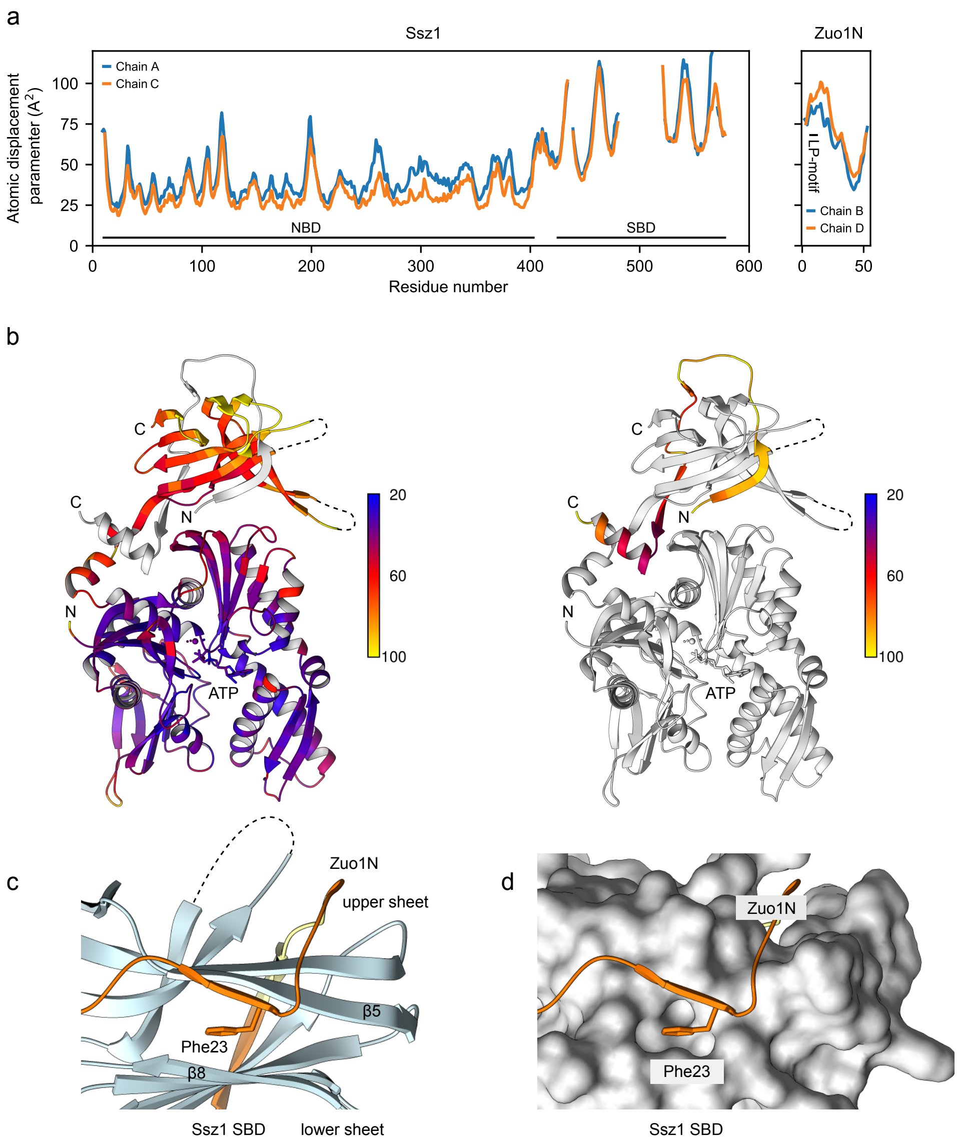

Supplementary Figure 3.

Analysis of Ssz1-SBD stabilization by Zuo1N.

(**a**) B-factor plot of the Ssz1-Zuo1N structure. The two heterodimers in the asymmetric unit are represented in blue (heterodimer 1: chain A and B) and in orange (heterodimer 2: chain C and D). The atomic displacement parameters (calculated with BAVERAGE in CCP4 [8] are plotted as a function of the residue numbers).

(**b**) Ribbon representation of Ssz1 (left) and Zuo1N (right) colored according to the atomic displacement parameters, scaled from 20 (blue) to 100 (yellow) $Å^2$. ATP is represented as sticks with the coordinated Mg^{2+} and waters as spheres.

(**c** and **d**) Stabilization of Ssz1-SBDβ by Zuo1 Phe23 stacking between the upper and lower β-sheets. Zuo1 is shown as ribbon representation (orange). Ssz1-SBDβ is shown as ribbon representation (cyan, **c**) or as surface representation (gray, **d**).

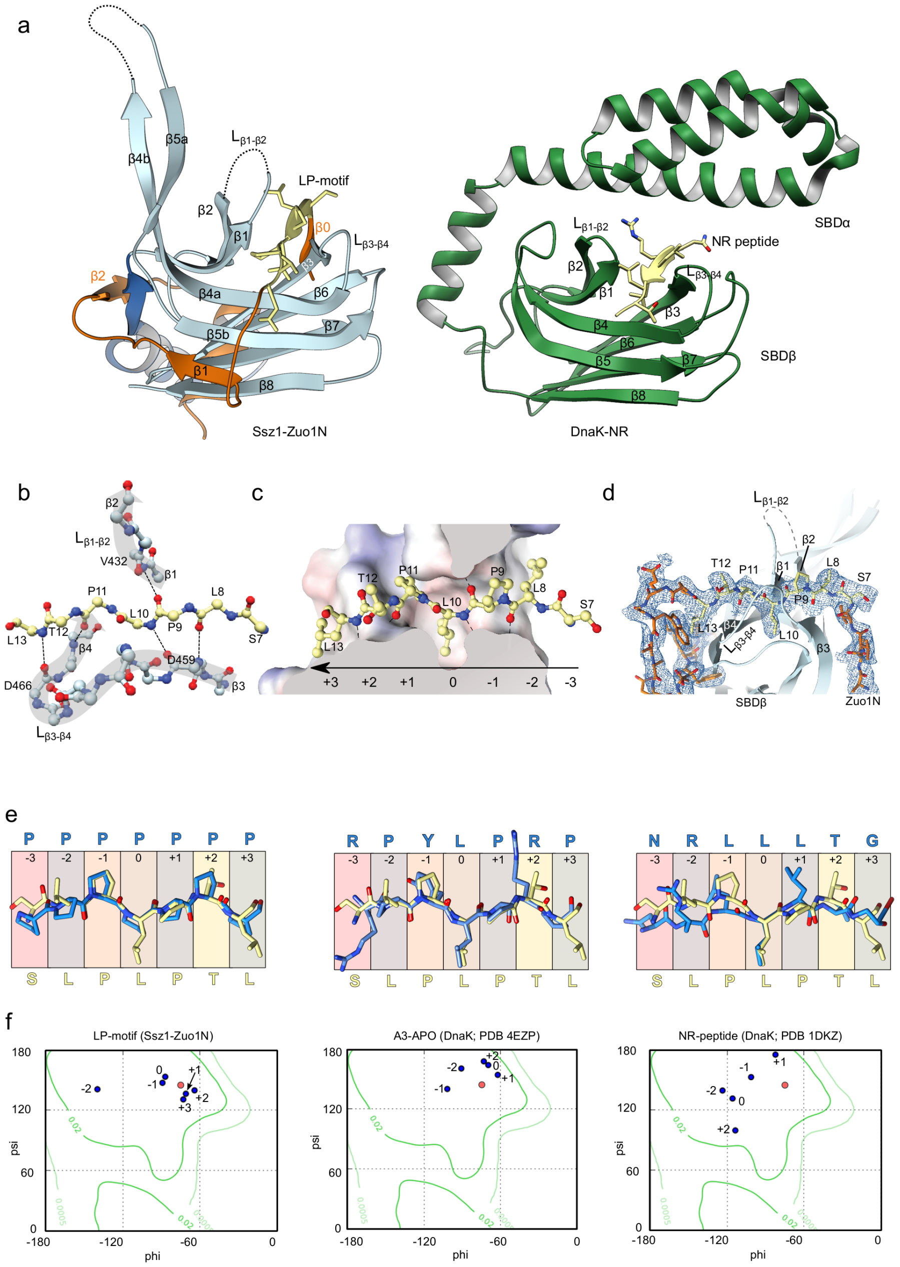

Supplementary Figure 4.

Details of Ssz1-Zuo1N substrate binding.

(**a**) Comparison of substrate binding to Ssz1-SBDβ on the left (this study) and DnaK-SBD on the right (PDB 1DKZ [9]). The coloring is as in **Fig. 2e**. Proteins are shown in ribbon representation. The LP-motif ([7]SLPLPTL[13]) of Zuo1 and the NR-peptide (NRLLLTG) are in yellow, side chains are in stick representation.

(**b**) Detailed view on the H-bonding (black discontinuous lines) between the Zuo1N LP-motif (yellow) and Ssz1-SBD β-strands (cyan). Only main chain atoms are shown, represented as ball-and-sticks.

(**c**) Surface representation of the Ssz1 substrate binding pocket colored by electrostatic surface potential from -5 to +5 kT/e (from red to blue), with the Zuo1N LP-motif (yellow) in ball-and-stick representation. H-bonds are indicated by dotted lines.

(**d**) Electron density map of Zuo1N (orange, with the LP-motif highlighted in yellow) represented as sticks. The Ssz1-SBD is in ribbon representation (cyan). The electron density map (2Fo-Fc) was contoured at 1σ.

(**e**) Comparison of the *Ct*Zuo1 LP-motif (bound to Ssz1) with an ideal PPII helix (left), with a proline-rich antimicrobial peptide (A3-APO) (middle, PDB 4EZP [10]), or with the NR-peptide (right, PDB 1DKZ[9]). The PPII helix was generated in UCSF Chimera [11]. The numbers indicate the position of each amino acid with respect to the Leu that occupies the center of the substrate binding pocket (position 0). The amino acid at each position is indicated by its one letter code in blue (ideal polyproline-II helix or NR-peptide) or in yellow (LP-motif).

(**f**) Ramachandran plot for the LP-motif (left), A3-APO (middle), or the NR-peptide (right) bound to their cognate SBD respectively. The ideal (φ,ψ) backbone dihedral angles of a PPII helix are indicated with a red dot. The numbers indicate the position of each residue with respect to the Leu at position 0.

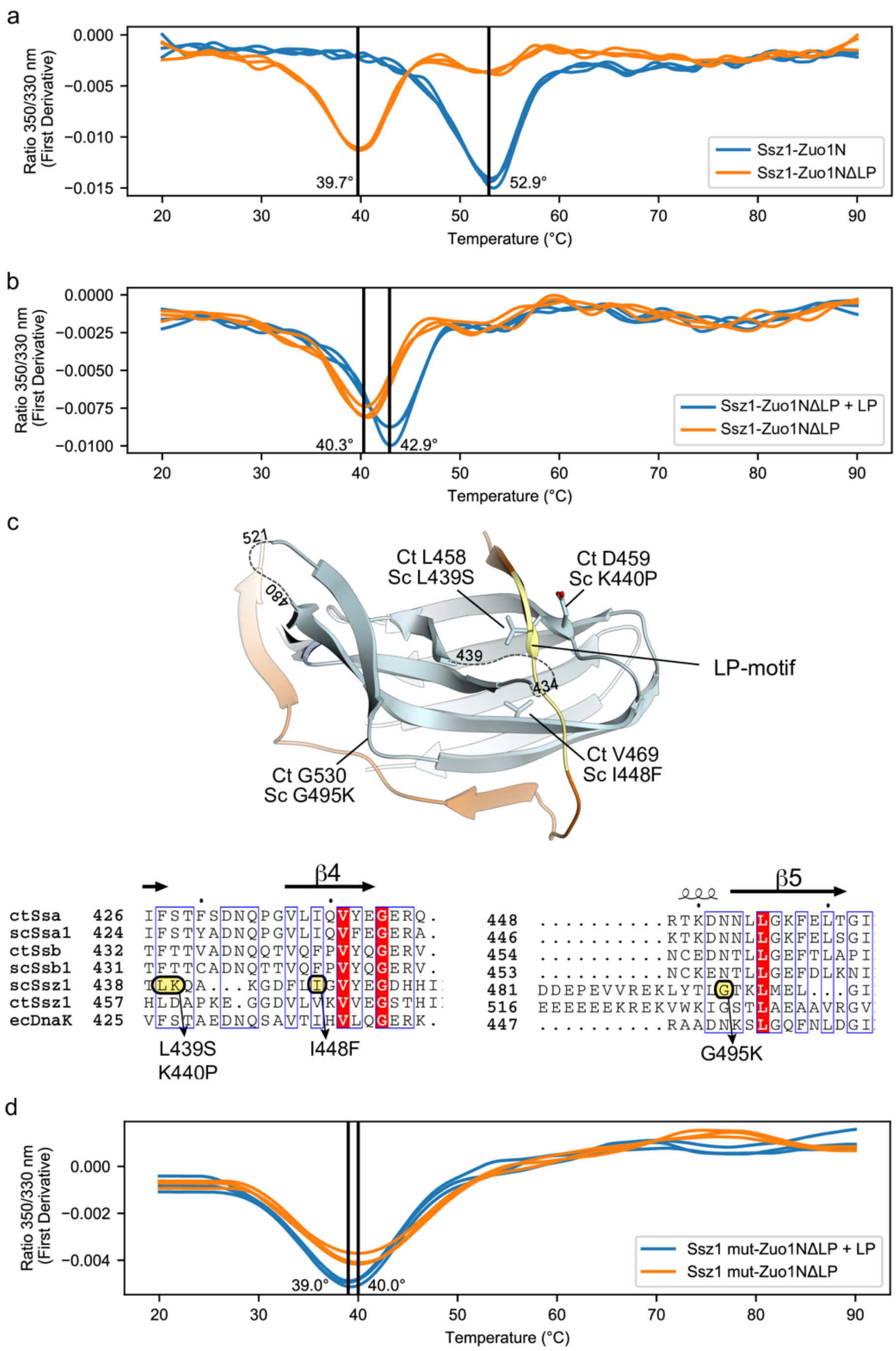

Supplementary Figure 5.

Thermostability profile of the *Sc*RAC core variants determined by nanoDSF.

(a-b) NanoDSF analysis of Ssz1-Zuo1N (blue) and Ssz1-Zuo1NΔLP (orange) in **a** and Ssz1-Zuo1NΔLP without the LP-peptide (orange) or with the LP-peptide (blue) in **b**. Three measurements are shown. Source data are provided as a Source Data file.

(**c**) Residues (L439S/K440P/I448F/G495K) mutated in Ssz1mut to block substrate binding to Ssz1-SBDβ (cyan) are in stick representation (upper panel). Zuo1N is shown in orange and the LP-motif in yellow. Multiple sequence alignment of Hsp70 homologs (lower panel) with the mutations highlighted in yellow. The alignment is colored as in **Fig. 2a**.

(**d**) NanoDSF analysis of Ssz1mut-Zuo1NΔLP without the LP-peptide (orange) or with the LP-peptide (blue). Three measurements are shown. Source data are provided as a Source Data file.

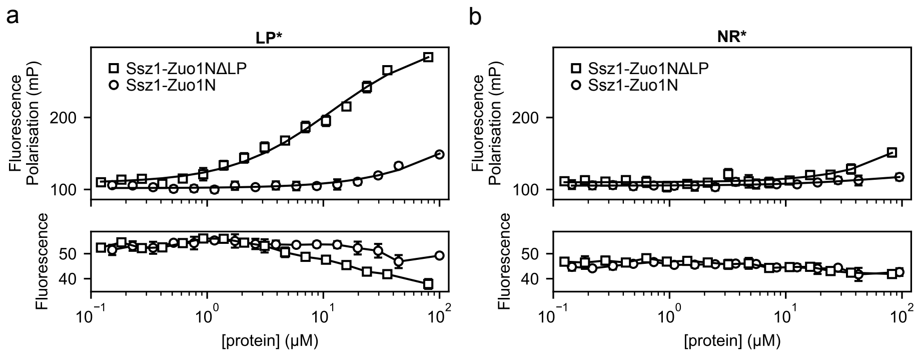

Supplementary Figure 6.

Analysis of substrate binding to *Sc*RAC core variants by fluorescence anisotropy.

(a) Binding curves of the fluorescently labeled LP-peptide or **(b)** NR-peptide to Ssz1-Zuo1N (circle) or Ssz1-Zuo1NDLP (square) are shown. The mean and standard deviation from three technical replicates is indicated. Source data are provided as a Source Data file.

Supplementary Table 1. Strains

Name	Genotype	Reference
S. cerevisiae		
MH272-3fα/a	*ura3 leu2 his3 trp1 ade2*	[12]
Δ*zuo1*	*zuo1::TRP1*	[13]
Δ*ssz1*	*ssz1::LEU2*<br>+ pYEPlac195-Zuo1	[14]
Δ*zuo1*Δ*ssz1*	*ssz1::LEU2 zuo1::TRP1*	[13]
Δ*ssb1*Δ*ssb2*	*ssb1::ADE2 ssb2::HIS3*	[15]
Δ*ssb1*Δ*ssb2*Δ*zuo1*Δ*ssz1*	*ssb1::ADE2 ssb2::ADE2 ssz1::LEU2 zuo1::TRP1*	[16]
mycSsb1	*ssb1::ADE2 ssb2::ADE2*<br>+ pCM190-mycSsb1	[1]
Ssb1-ΔC23	*ssb1::ADE2 ssb2::ADE2*<br>+ pCM190-mycSsb1-ΔC23	[1]
Δ*ssb1*Δ*ssb2*Δ*ssz1*	*ssb1::KAN ssb2::HIS3 ssz1::LEU2*<br>+ pYEPlac195-Zuo1	[14]
Δ*ssb1*Δ*ssb2*Δ*ssz1*	*ssb1::ADE2 ssb2::ADE2 ssz1::LEU2*	this study
RAC-ΔN8	*zuo1::TRP1*<br>+ pRS315-Zuo1-ΔN8	this study
RAC-ΔN16	*zuo1::TRP1*<br>+ pRS315-Zuo1-ΔN16	[7]
RAC-ΔN49	*zuo1::TRP1*<br>+ pRS315-Zuo1-ΔN49	[7]
Δ*ssb1*Δ*ssb2* RAC-ΔN8	*ssb1::ADE2 ssb2::ADE2 zuo1::TRP1*<br>+ pRS315-Zuo1-ΔN8	this study
Δ*ssb1*Δ*ssb2* RAC-ΔN16	*ssb1::ADE2 ssb2::ADE2 zuo1::TRP1*<br>+ pRS315-Zuo1-ΔN16	this study
Δ*ssb1*Δ*ssb2* RAC-ΔN49	*ssb1::ADE2 ssb2::HIS3 zuo1::TRP1*<br>+ pRS315-Zuo1-ΔN49	this study
Δ*ssb1*Δ*ssb2* RAC-Ssz1mut	*ssb1::ADE2 ssb2::ADE2 ssz1::LEU2* + pYCPlac33-Ssz1mut	this study
Δ*ssb1*Δ*ssb2* Zuo1-ΔN8 Ssz1mut	*ssb1::ADE2 ssb2::ADE2 ssz1::LEU2 zuo1::TRP1*<br>+pRS423-Zuo1-ΔN8 + pYCPlac33-Ssz1mut	this study
E. coli		
BL21(DE3) Rosetta2	F^- *ompT hsd*S_B(rB^- mB^-) *gal dcm* (DE3) pRARE2 (Cam^R)	Novagen

Supplementary Table 2. Plasmids

**Name**	**Description**	**Reference**
pCM190-mycSsb1	2µ, *URA3,* myc*ScSSB1*	[1]
pCM190-mycSsb1-ΔC23	2µ, *URA3,* myc*ScSSB1*-ΔC23	[1]
pYEPlac195-Zuo1	2µ, *URA3, ScZUO1*	[14]
pRS315-Zuo1	CEN, *LEU2, ScZUO1*	[7]
pRS315-Zuo1-ΔN8	CEN, *LEU2, ScZUO1*-ΔN8	this study
pRS315-Zuo1-ΔN16	CEN, *LEU2, ScZUO1*-ΔN16	[7]
pRS315-Zuo1-ΔN49	CEN, *LEU2, ScZUO1*-ΔN49	[7]
pR423-Zuo1-ΔN8	CEN, *HIS3, ScZUO1*-ΔN8	this study
pYCPlac33-Ssz1	CEN, URA3 *Sc* Ssz1	[5]
pYCPlac33-Ssz1mut	CEN, *URA3*, *ScSSZ1* L439S/K440P/I448F/G495K	this study
pRS315-*Sc*Ssz1	CEN, *LEU2, ScSSZ1*	this study
pET16b-His$_6$-MBP	Amp^R, His$_6$-MBP	G. Stier
pET24d-His$_6$-SUMO	Kan^R, His$_6$-SUMO	G. Stier
pET16b-His$_6$-MBP-GSGSGS-TEV-*Ct*Zuo1	Amp^R, His$_6$-MBP-GSGSGS-TEV-*CtZUO1*	[7]
pET16b-His$_6$-MBP-3C-*Ct*Zuo1N	Amp^R, *CtZUO1* residues 1 to 60	this study
pET24d-His$_6$-SUMO-Strep-*Ct*Ssz1	Kan^{R,} His$_6$-SUMO-Strep-*CtSSZ1*	[7]
pET16b-His$_6$-MBP-3C-*Sc*Zuo1N	Amp^R, *ScZUO1* residues 1 to 50	this study
pET16b-His$_6$-MBP-3C-*Sc*Zuo1NΔLP	Amp^R, *ScZUO1* residues 10 to 50	this study
pET24d-His$_6$-SUMO-*Sc*Ssz1	Kan^R, His$_6$-SUMO-*ScSSZ1*	this study
pET24d-His$_6$-SUMO-*Sc*Ssz1mut	Kan^R, *ScSSZ1* L439S/K440P/I448F/G495K	this study

Supplementary Table 3. Primers

Primers for cloning of	Sense	Antisense
**pYCPlac33-Ssz1mut**<br>introduction of: 439S440P448F	5’TTAAAGTTAGCCGCGGAAGATT ACATCG	5’TCGATGTGATGGTCACCTT CGTAAACACCAAaCAAGAAATCACCC TTGGCTTGTggCgAAGTCAATTTCTTT TGTACAGGG
introduction of: Ssz1495K	5’CTATACACTTTGAAGACCAA GTTGATGG	5’CCATCAACTTGGTCTTCAAA GTGTATAG
**pET16b-His$_6$-MBP-3C-*Ct*Zuo1N**	5’GCTTAACCATGGACGCGAC CG	5’TTAATTGGATCCTTAGGA GAAGGTACGCTTGTGACG
**pET16b-His$_6$-MBP-3C-*Sc*Zuo1N**	5’AATTAACCATGGTTTCTTTA CCTACCCTAACCTCAG	5’GCTTAAGGATCCTTAAGA CCAGGTGTGGTTTCTCAAAG

**pET24d-His$_6$-SUMO-*Sc*Ssz1**	5'AATTAACCATGGGCTCCTCT CCAGTGATTGGTATCAC	5'AATTAAGGATCCTTATAAT TCACCCTTTACAGCATTACCAG
**pRS315-*Sc*Ssz1**	5'TTAATTTCTAGAGAAGTTGT AATGGAAATGAACAGAAG	5'TTAATTGGATCCACCCAAC CCAAGAATATGAATAGTAC
**His$_6$-MBP-3C**	5'CACTATAGGGGAATTGTGA GC	5'CGCTGCCCATGGCCGGGC CCTGAAACAGCACTTCCAGAGAACCA CTGCCAGATCCCG
**pET16b-His$_6$-MBP-3C-*Sc*Zuo1NΔLP**	5'GAAGTGCTGTTTCAGGGCC CGGACATCACTGTTGAAGTCAAC AG	5'CTGTTGACTTCAACAGTGA TGTCCGGGCCCTGAAACAGCACTTC
**pRS315-*Sc*Zuo1-ΔN8**	5'CATATACCAACAAGAGTAAC GATGTCAGACATCACTGTTGAAG TCAAC	5'GTTGACTTCAACAGTGATG TCTGACATCGTTACTCTTGTTGGTAT ATG

Supplementary Methods.

Media and culture conditions. Yeast strains were grown to log phase on 1% yeast extract, 2% peptone and 2% dextrose (YPD) or in glucose-containing minimal media. Growth defects were analysed by spotting 10-fold serial dilutions containing the same number of cells onto YPD plates. When indicated, plates were supplemented with paromomycin. Total yeast extract for immunoblot analysis was prepared as described [17].

Supplementary References.

1. Gumiero, A. *et al.* Interaction of the cotranslational Hsp70 Ssb with ribosomal proteins and rRNA depends on its lid domain. *Nat. Commun.* **7,** 1-12 (2016).
2. Peisker, K., Chiabudini, M. & Rospert, S. The ribosome-bound Hsp70 homolog Ssb of Saccharomyces cerevisiae. *Biochim. Biophys. Acta* **1803,** 662-672 (2010).
3. Zhang, Y., Sinning, I. & Rospert, S. Two chaperones locked in an embrace: Structure and function of the ribosome-associated complex RAC. *Nat. Struct. Mol. Biol.* **24,** 611-619 (2017).
4. Hundley, H. *et al.* The in vivo function of the ribosome-associated Hsp70, Ssz1, does not require its putative peptide-binding domain. *Proc. Natl. Acad. Sci. U S A* **99,** 4203-4208 (2002).
5. Conz, C. *et al.* Functional characterization of the atypical Hsp70 subunit of yeast ribosome-associated complex. *J. Biol. Chem.* **282,** 33977-33984 (2007).
6. Hanebuth, M. A. *et al.* Multivalent contacts of the Hsp70 Ssb contribute to its architecture on ribosomes and nascent chain interaction. *Nat. Commun.* **7,** 13695 (2016).
7. Weyer, F. A., Gumiero, A., Gese, G. V., Lapouge, K. & Sinning, I. Structural insights into a unique Hsp70-Hsp40 interaction in the eukaryotic ribosome-associated complex. *Nat. Struct. Mol. Biol.* **24,** 144-151 (2017).
8. Winn, M. D. *et al.* Overview of the CCP4 suite and current developments. *Acta Crystallographica. Section D, Biological Crystallography* **67,** 235-242 (2011).
9. Zhu, X. *et al.* Structural analysis of substrate binding by the molecular chaperone DnaK. *Science* **272,** 1606-1614 (1996).
10. Zahn, M. *et al.* Structural studies on the forward and reverse binding modes of peptides to the chaperone DnaK. *J. Mol. Biol.* **425,** 2463-2479 (2013).

11. Pettersen, E. F. *et al.* UCSF Chimera--a visualization system for exploratory research and analysis. *J. Comput. Chem.* **25,** 1605-1612 (2004).
12. Heitman, J., Movva, N. R., Hiestand, P. C. & Hall, M. N. FK 506-binding protein proline rotamase is a target for the immunosuppressive agent FK 506 in Saccharomyces cerevisiae. *Proc. Natl. Acad. Sci. U S A* **88,** 1948-1952 (1991).
13. Gautschi, M. *et al.* RAC, a stable ribosome-associated complex in yeast formed by the DnaK-DnaJ homologs Ssz1p and zuotin. *Proc. Natl. Acad. Sci. U S A* **98,** 3762-3767 (2001).
14. Gautschi, M., Mun, A., Ross, S. & Rospert, S. A functional chaperone triad on the yeast ribosome. *Proc. Natl. Acad. Sci. U S A* **99,** 4209-4214 (2002).
15. Rakwalska, M. & Rospert, S. The Ribosome-Bound Chaperones RAC and Ssb1/2p are Required for Accurate Translation in Saccharomyces cerevisiae. *Mol. Cell. Biol.* **24,** 9186-9197 (2004).
16. Jaiswal, H. *et al.* The chaperone network connected to human ribosome-associated complex (mRAC). *Mol. Cell. Biol.* **31,** 1160-1173 (2011).
17. Yaffe, M. P. & Schatz, G. Two nuclear mutations that block mitochondrial protein import in yeast. *Proc. Natl. Acad. Sci. U S A* **81,** 4819-4823 (1984).
