# Supplementary material for: The ribosome-associated complex RAC serves in a relay that directs nascent chains to Ssb
Source: Nat Commun. 2020 Mar 20;11:1504. doi: 10.1038/s41467-020-15313-w (PMC7083937; doi:10.1038/s41467-020-15313-w)
Supplement: Supplementary file 3 — Reporting Summary [file 41467_2020_15313_MOESM3_ESM.pdf]

nature research

Corresponding author(s): Irmgard Sinning, Sabine Rospert

Last updated by author(s): February 25, 2020

Reporting Summary

Nature Research wishes to improve the reproducibility of the work that we publish. This form provides structure for consistency and transparency in reporting. For further information on Nature Research policies, see Authors & Referees and the Editorial Policy Checklist .

Statistics

For all statistical analyses, confirm that the following items are present in the figure legend, table legend, main text, or Methods section.

n/a	Confirmed	
☐	☒	The exact sample size (*n*) for each experimental group/condition, given as a discrete number and unit of measurement
☐	☒	A statement on whether measurements were taken from distinct samples or whether the same sample was measured repeatedly
☐	☒	The statistical test(s) used AND whether they are one- or two-sided<br>*Only common tests should be described solely by name; describe more complex techniques in the Methods section.*
☒	☐	A description of all covariates tested
☒	☐	A description of any assumptions or corrections, such as tests of normality and adjustment for multiple comparisons
☐	☒	A full description of the statistical parameters including central tendency (e.g. means) or other basic estimates (e.g. regression coefficient) AND variation (e.g. standard deviation) or associated estimates of uncertainty (e.g. confidence intervals)
☒	☐	For null hypothesis testing, the test statistic (e.g. *F*, *t*, *r*) with confidence intervals, effect sizes, degrees of freedom and *P* value noted<br>*Give P values as exact values whenever suitable.*
☒	☐	For Bayesian analysis, information on the choice of priors and Markov chain Monte Carlo settings
☒	☐	For hierarchical and complex designs, identification of the appropriate level for tests and full reporting of outcomes
☒	☐	Estimates of effect sizes (e.g. Cohen's *d*, Pearson's *r*), indicating how they were calculated

Our web collection on statistics for biologists contains articles on many of the points above.

Software and code

Policy information about availability of computer code

Data collection	Diffraction data were measured under cryogenic conditions (100 K; Oxford Cryosystems Cryostream) at the European Synchrotron Radiation Facility (ESRF, Grenoble) beamline id30a3.
Data analysis	Data were processed with XDS. Phases were obtained by molecular replacement using PHASER in the CCP4I2 software package. Iterative model building and refinement were done with COOT and phenix.refine . The validation was performed using EDSTATS and MOLPROBITY included in CCP4I2. UCSF chimera was used for figure preparation. Sequence alignments were performed using Clustal Omega and visualized with ESPript 3.0.<br>In addition, the following software was used in this study:<br>Python version 3.6; Graphpad Prism version 6.07; AIDA ImageAnalyzer version 4.27.

For manuscripts utilizing custom algorithms or software that are central to the research but not yet described in published literature, software must be made available to editors/reviewers. We strongly encourage code deposition in a community repository (e.g. GitHub). See the Nature Research guidelines for submitting code & software for further information.

Data

Policy information about availability of data

All manuscripts must include a data availability statement. This statement should provide the following information, where applicable:

- Accession codes, unique identifiers, or web links for publicly available datasets
- A list of figures that have associated raw data
- A description of any restrictions on data availability

Accession codes: Coordinates and structure factors have been deposited in the Protein Data Bank under accession code 6SR6.
The PDB datasets 5MB9, 1DKZ and 4EZP have been used in this study.
The source data underlying Figures 1c-f, 2b–e, 3b-f, and Supplementary Figures 1a-j, 5a-c, and 6a,b are provided as a Source Data file.
Other data that support the findings of this study are available from the corresponding authors on reasonable request.

Field-specific reporting

Please select the one below that is the best fit for your research. If you are not sure, read the appropriate sections before making your selection.

☒ Life sciences ☐ Behavioural & social sciences ☐ Ecological, evolutionary & environmental sciences

For a reference copy of the document with all sections, see nature.com/documents/nr-reporting-summary-flat.pdf

Life sciences study design

All studies must disclose on these points even when the disclosure is negative.

Sample size	n=1 to 3
Data exclusions	No data were excluded from the analysis.
Replication	2 to 3 replicates were performed.
Randomization	This study did not involve experimental groups.
Blinding	No blind experiments were performed.

Reporting for specific materials, systems and methods

We require information from authors about some types of materials, experimental systems and methods used in many studies. Here, indicate whether each material, system or method listed is relevant to your study. If you are not sure if a list item applies to your research, read the appropriate section before selecting a response.

Materials & experimental systems

n/a	Involved in the study	
☐	☒	Antibodies
☒	☐	Eukaryotic cell lines
☒	☐	Palaeontology
☒	☐	Animals and other organisms
☒	☐	Human research participants
☒	☐	Clinical data

Methods

n/a	Involved in the study	
☒	☐	ChIP-seq
☒	☐	Flow cytometry
☒	☐	MRI-based neuroimaging

Antibodies

Antibodies used	Antibodies for immunoblotting and denaturing immunoprecipitation reactions are rabbit polyclonal antibodies (Rospert lab antibody collection). Antibody dilutions were as follows: alpha-Ssb 1:5.000, alpha-Ssz1 1:20.000, alpha-Zuo1 1:40.000, alpha-Sse1 1:20.000, alpha-Rps9 1:8.000, alpha-Rpl4 1:20.000, and alpha-Pgk1 1:20.000.<br>Horseradish-conjugated goat anti-rabbit IgG (Pierce catalogue number 31460, 1:10.000) was employed as a secondary antibody.
Validation	Raue, U., Oellerer, S. & Rospert, S. Association of protein biogenesis factors at the yeast ribosomal tunnel exit is affected by the translational status and nascent polypeptide sequence. J. Biol. Chem. 282, 7809–7816 (2007).
